# Supplementary material for: Prostate MRI added to CAPRA, MSKCC and Partin cancer nomograms significantly enhances the prediction of adverse findings and biochemical recurrence after radical prostatectomy
Source: PLoS One. 2020 Jul 9;15(7):e0235779. doi: 10.1371/journal.pone.0235779 (PMC7347171; doi:10.1371/journal.pone.0235779)
Supplement: S1 Table — a) Comparison of mpMRI, clinical and pathological staging. Distribution of lymph node finding; 1 b) Distribution of PI-RADS and prostatectomy grading. PI-RADS score in multiparametric MRI and Gleason Grade Group in surgical specimen for ROI1. (DOCX) [file pone.0235779.s001.docx]

| Supplementary table 1a. Comparison of mpMRI, clinical and pathological staging. Distribution of lymph node findings. (n=348) | | | | |
| --- | --- | --- | --- | --- |
|  | **mpMRI** | | **cT** | **pTN** |
| Tumor Staging. No. (%) | 387 (100) | | | |
| T1c (PI-RADS < 3) | 39 (10) | | 241 (62.4) | - |
| T2a | 126 (32.5) | | 29 (7.5) | 35 (9) |
| T2b | 4 (1) | | 11 (2.8) | - |
| T2c | 62 (16) | | 33 (8.5) | 186 (48.1) |
| T3a | 102 (26.4) | | 66 (17) | 111 (28.7) |
| T3b | 36 (9.3) | | 5 (1.3) | 54 (14) |
| T4 | 18 (4.7) | | 2 (0.5) | 1 (0.3) |
|  |  | |  |  |
| Lymph node staging. No. (%) |  | |  |  |
| Lymph node dissection | 188 (48.6) | |  |  |
| Positive lymph nodes, N+ | 11 (2.8) | |  | 36 (19.1) |
| pTN: pathological staging. cT: clinical staging | | | | |
|  | | | | |
| Supplementary table 1b. Distribution of PI-RADS and Prostatectomy Grading. PI-RADS score in mpMRI and GGG in PAD for ROI1. (n=348) | | | | |
|  | | **Result (SD or % of total)** | | |
| No. of lesions per case, mean (SD) | | 1.34 (0.75) | | |
| Missing | | 1 (0.03) | | |
| PI-RADS < 3 | | 40 (10.4) | | |
| PI-RADS 3 | | 24 (6.2) | | |
| PI-RADS 4 | | 41 (10.6) | | |
| PI-RADS 5 | | 281 (72.6) | | |
|  | |  | | |
| No. of lesions per case, mean (SD) | | 2.19 (1.12) | | |
| GGG1 | | 14 (3.6) | | |
| GGG2 | | 149 (38.5) | | |
| GGG3 | | 169 (43.7) | | |
| GGG4 | | 13 (3.4) | | |
| GGG5 | | 42 (10.9) | | |
| ROI: region of interest; GGG: Gleason Grade Group; PAD: pathological diagnosis in RP | | | | |
