# Supplementary material for: Prostate MRI added to CAPRA, MSKCC and Partin cancer nomograms significantly enhances the prediction of adverse findings and biochemical recurrence after radical prostatectomy
Source: PLoS One. 2020 Jul 9;15(7):e0235779. doi: 10.1371/journal.pone.0235779 (PMC7347171; doi:10.1371/journal.pone.0235779)
Supplement: S2 Table — (DOCX) [file pone.0235779.s002.docx]

| **Supplementary table 2. Cox proportional hazard model summaries: prediction of biochemical recurrence.** | | | | | | | | | |
| --- | --- | --- | --- | --- | --- | --- | --- | --- | --- |
|  | | | | | | | |  | |
| **Model without mpMRI parameters** | | | | | **Model with mpMRI parameters** | | | | |
| **Estimate** | | **HR (95% CI)** | ***p* value** | | **Estimate** | | **HR (95% CI)** | | ***p* value** |
|  |  |  | |  | **MRI** |  |  | |  |
|  |  |  | |  | MRI ANY | 0.826 | 2.284 (1.402-3.723) | | **<0.001** |
|  |  |  | |  |  |  |  | |  |
| **CAPRA** |  |  | |  | **CAPRA + MRI** |  |  | |  |
| CAPRASUM | 0.399 | 1.49 (1.307-1.699) | | **<0.001** | CAPRASUM | 0.373 | 1.452 (1.272-1.658) | | **<0.001** |
|  |  |  | |  | MRI ANY | 0.479 | 1.614 (0.967-2.693) | | 0.067 |
|  |  |  | |  |  |  |  | |  |
| **MSKCC** |  |  | |  | **MSKCC + MRI** |  |  | |  |
| MSKCC 3-year  survival % | -0.0291 | 0.971 (0.963-0.98) | | **<0.001** | MSKCC 3-year survival % | -0.0268 | 0.974 (0.965-0.982) | | **<0.001** |
|  |  |  | |  | MRI ANY | 0.460 | 1.584 (0.949-2.645) | | 0.079 |
|  |  |  | |  |  |  |  | |  |
| **CLINICAL** |  |  | |  | **CLINICAL + MRI** |  |  | |  |
| PSAPre | 0.0598 | 1.062 (1.036-1.088) | | **<0.001** | PSAPre | 0.0594 | 1.061 (1.036-1.087) | | **<0.001** |
| Age | 0.041 | 1.042 (1.001-1.084) | | **0.044** | Age | 0.0402 | 1.041 (1.001-1.083) | | **0.047** |
| GGG ≥ 3 | 1.286 | 3.619 (1.762-7.433) | | **<0.001** | GGG ≥ 3 | 1.269 | 3.559 (1.734-7.302) | | **<0.001** |
| cT ≥ 3 | 0.906 | 2.474 (1.454-4.21) | | **<0.001** | cT ≥ 3 | 0.764 | 2.146 (1.205-3.822) | | **0.010** |
| PSAPre x GGG >= 3 | -0.043 |  | | **0.002** | PSAPre x  GGG >= 3 | -0.0441 |  | | **0.002** |
|  |  |  | |  | MRI ANY | 0.338 | 1.402 (0.809-2.43) | | 0.229 |
| MRI ANY: mpMRI suggesting extraprostatic extension, seminal vesicle invasion or lymph node involvement. OC: organ-confined. cT: clinical stage | | | | | | | | | |
