# Supplementary figures and images for: Prostate MRI added to CAPRA, MSKCC and Partin cancer nomograms significantly enhances the prediction of adverse findings and biochemical recurrence after radical prostatectomy
Source: PLoS One. 2020 Jul 9;15(7):e0235779. doi: 10.1371/journal.pone.0235779 (PMC7347171; doi:10.1371/journal.pone.0235779)

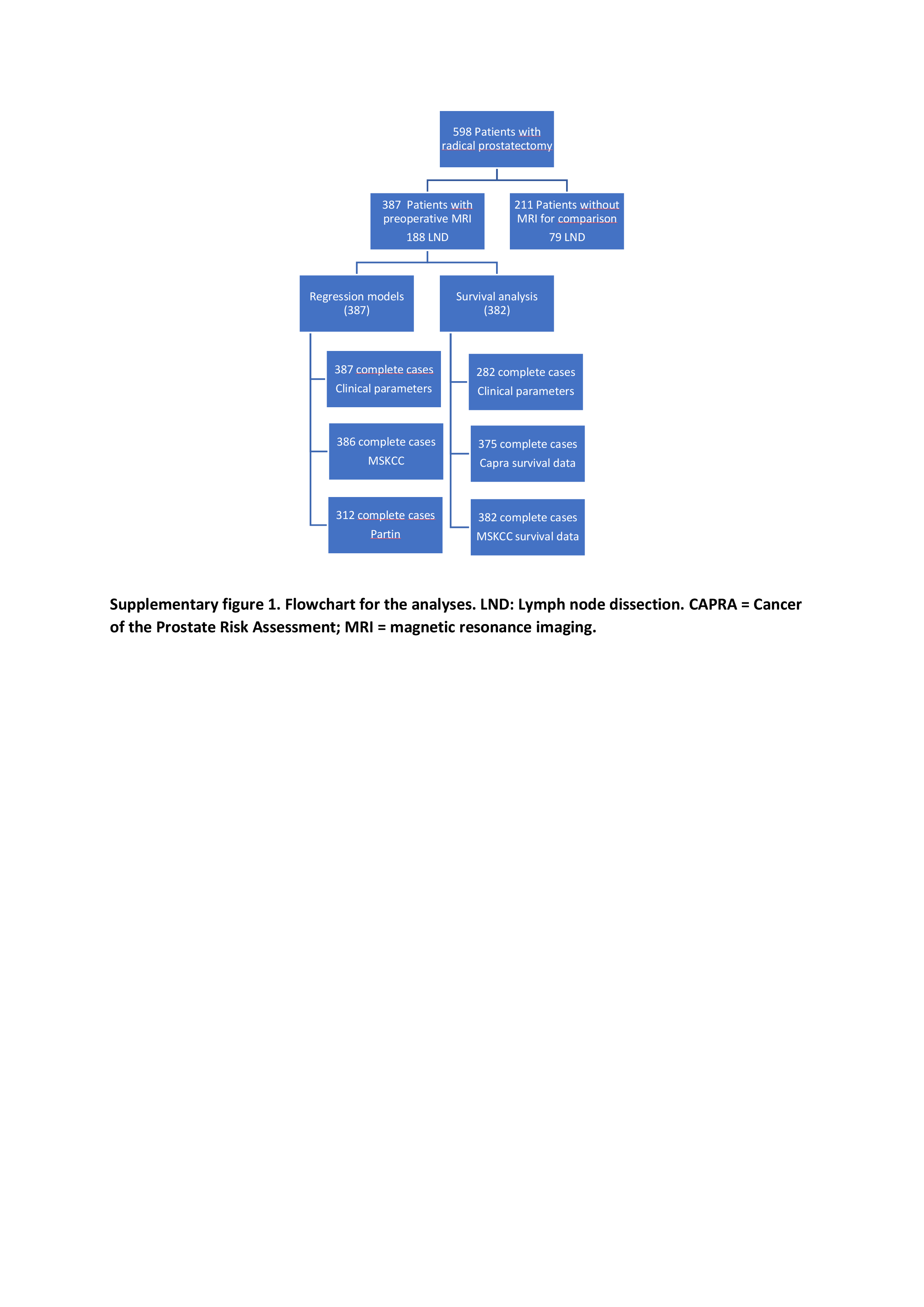

Supplement: S1 Fig — LND: Lymph node dissection. CAPRA = Cancer of the Prostate Risk Assessment; MRI = magnetic resonance imaging. (TIFF) [file pone.0235779.s003.tiff]

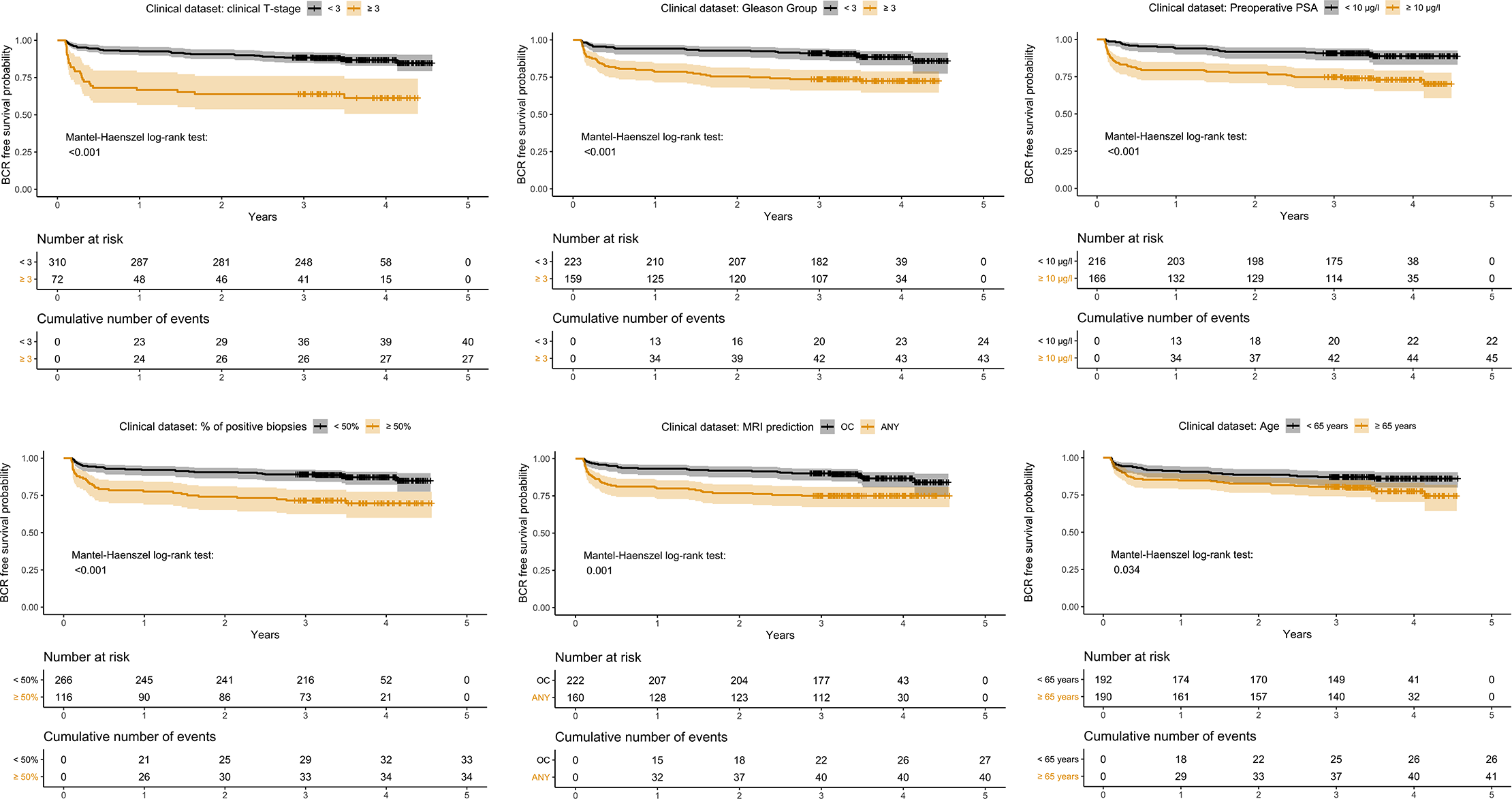

Supplement: S2 Fig — BCR free survival probability–Kaplan-Meier survival analysis for clinical parameters: a) clinical T-stage equal or higher than 3 (follow-up data for clinical T-stage missing for one patient); b) Gleason Grade Group equal or higher than 3; c) preoperative PSA equal or higher than 10 μg/l; d) percentage of positive biopsies equal or higher than 50%; e) MRI prediction for any adverse finding; f) age at operation equal or higher than 65 years. (TIF) [file pone.0235779.s004.tif]

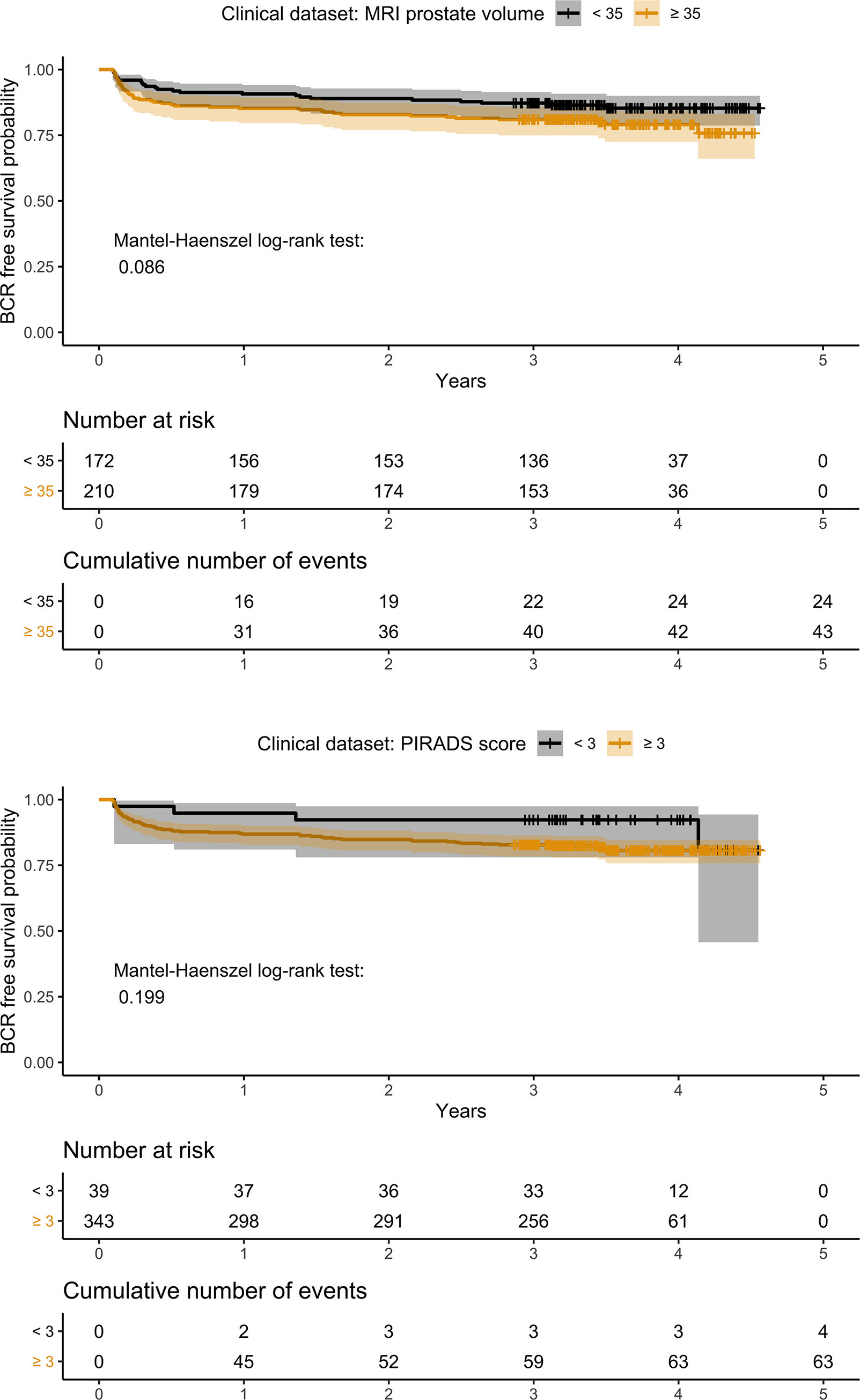

Supplement: S3 Fig — BCR free survival probability–Kaplan-Meier survival analysis for clinical parameters: a) mpMRI prostate volume (> 35 cc); b) PI-RADS score higher than two. (TIF) [file pone.0235779.s005.tif]

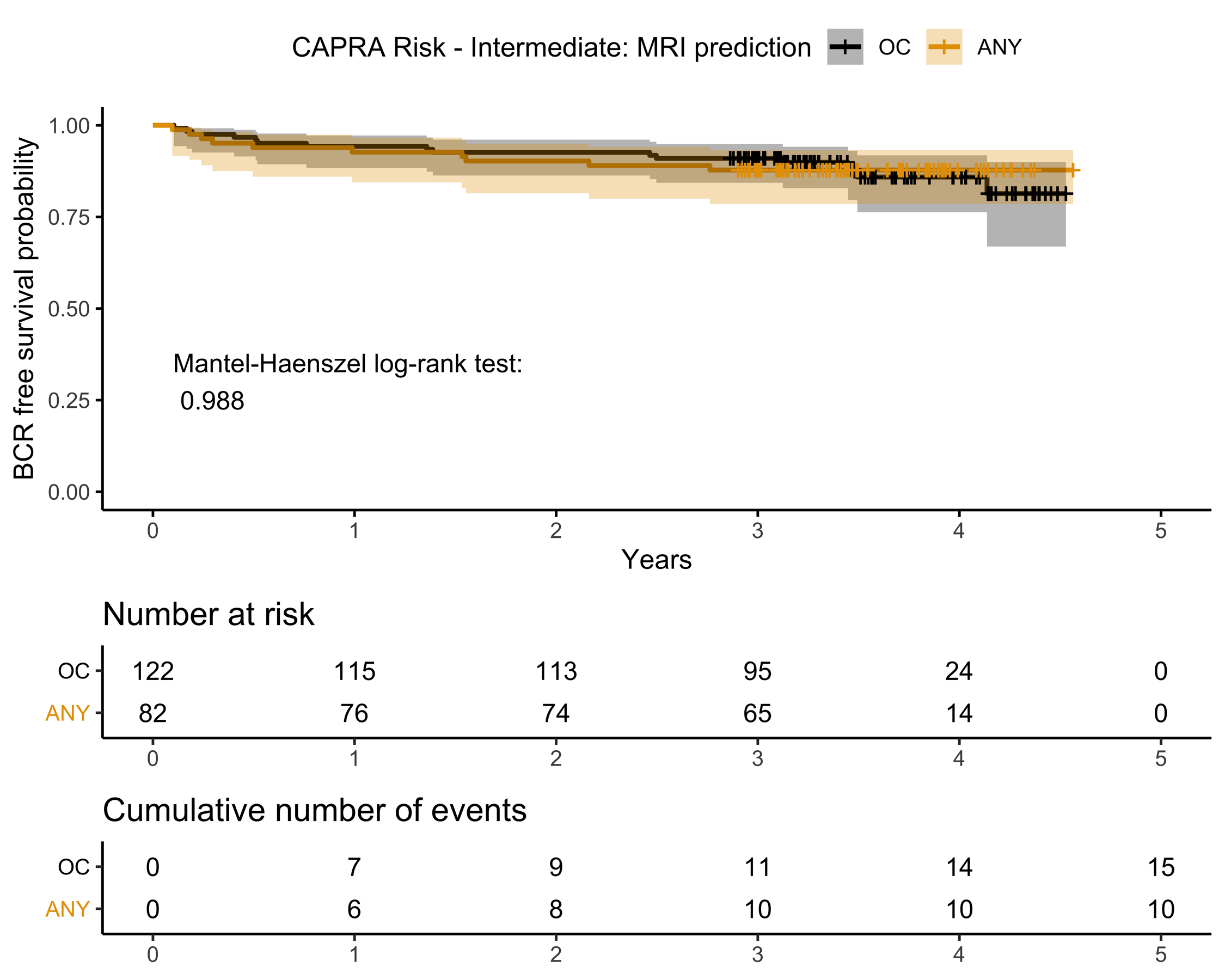

Supplement: S4 Fig — (TIFF) [file pone.0235779.s006.tiff]

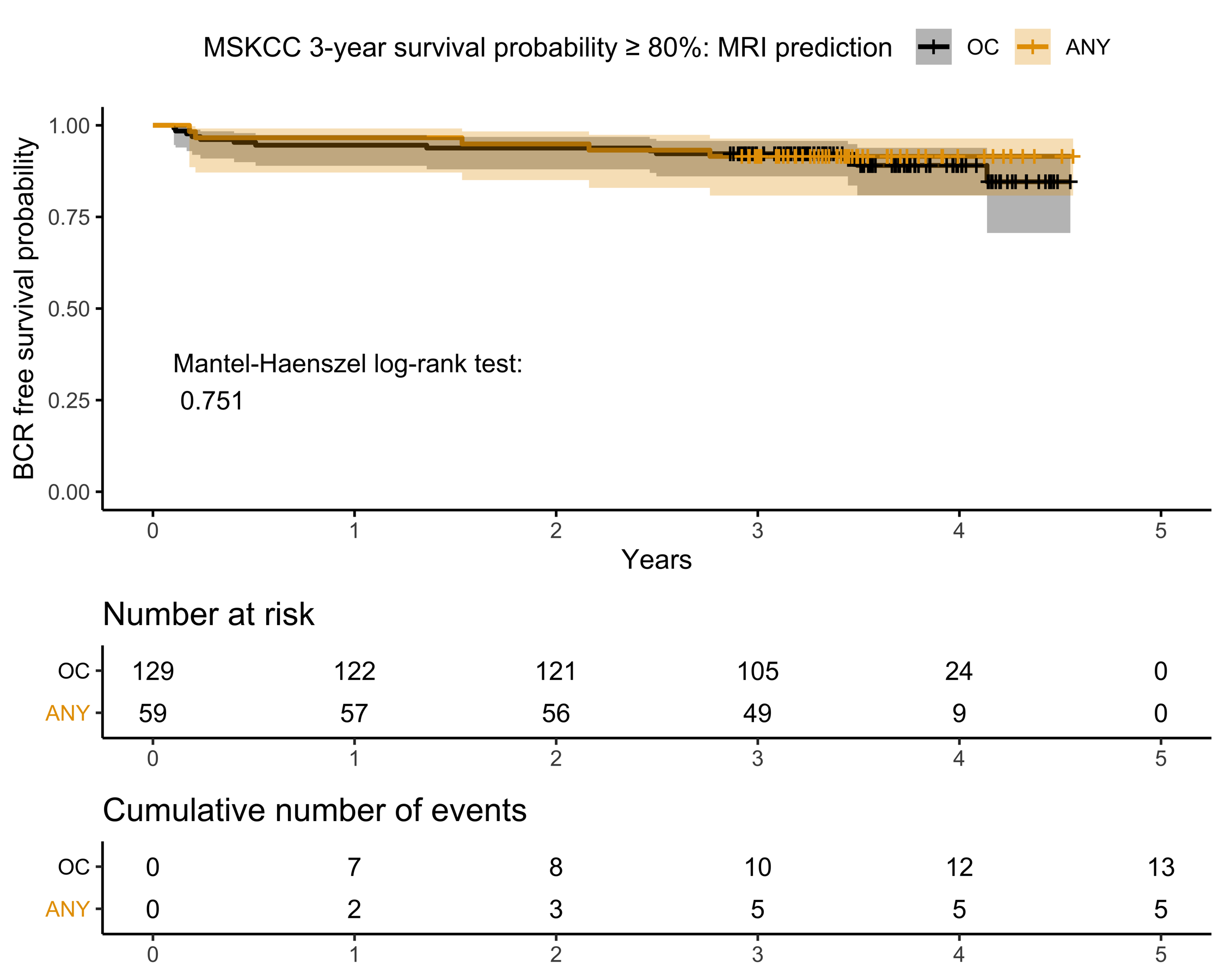

Supplement: S5 Fig — (TIFF) [file pone.0235779.s007.tiff]
